# Evaluation of the value of ENI in radiotherapy for cervical and upper thoracic esophageal cancer: a retrospective analysis

**DOI:** 10.1186/s13014-014-0232-4

**Published:** 2014-10-25

**Authors:** Mina Liu, Kuaile Zhao, Yun Chen, Guo-Liang Jiang

**Affiliations:** Department of Radiation Oncology, Shanghai Chest Hospital, Shanghai Jiao tong University, Shanghai, 200030 China; Department of Radiation Oncology, Fudan University Shanghai Cancer Center, 270 Dong An Road, Shanghai, 200032 China; Department of Oncology, Shanghai Medical College, Fudan University, 270 Dong An Road, Shanghai, 200032 China

**Keywords:** Esophageal squamous cell carcinoma, Radiotherapy, Elective nodes irradiation, Cervical failures

## Abstract

**Background:**

A retrospective study to compare the failure patterns and effects of elective nodal irradiation (ENI) or involved field irradiation (IFI) for cervical and upper thoracic esophageal squamous cell carcinoma (SCC) patients.

**Methods:**

One hundred and sixty nine patients with the cervical and upper thoracic esophageal SCC were analyzed retrospectively; 99 patients (59%) underwent IFI and 70 patients (41%) received ENI. We defined “Out-PTVifi in-PTVeni metastasis” as lymph node metastasis occurring in the cervical prophylactic field of PTVeni thus out of PTVifi.

**Results:**

Out-PTVifi in-PTVeni cervical node metastasis occurred in 8% of patients in the IFI group, all within 2 years after treatment. However, it occurred in 10% of patients in the ENI group, and these failures happened gradually since one year after treatments. No difference was found in OS and the incidences of Grade ≥ 3 treatment-related esophageal and lung toxicities between the two groups.

**Conclusions:**

ENI for cervical and upper thoracic esophageal SCC patients did not bring longer OS and better long-term control of cervical lymph nodes. Although ENI might delay cervical nodes progression in elective field; it could not decrease the incidence of these failures.

## Background

Esophageal carcinoma is one of the most common cancers in the world with an estimated 482,300 new cases and 406,800 deaths yearly worldwide [1]. Definitive chemoradiotherapy is widely used as the standard of care [2-4]. Whether ENI should be performed has always been a controversy, especially for cervical and upper thoracic esophageal squamous cell carcinoma (SCC). We retrospectively studied different failure patterns for patients who had ENI and who had not, investigated the effects for them.

## Materials and methods

### Patient selection criteria

Patients who met the following criteria were included for this study: (1) confirmation of esophageal SCC by histology or cytology; (2) no prior therapy (what therapy?); (3) no previous malignancies; (4) complete clinical data; (5) tumors proved to be located in cervical or upper thoracic esophagus by endoscopy. Patients who had been treated with surgery were excluded. A total of 350 consecutive patients with esophageal cancer were treated with definitive chemoradiotherapy at a single institution (Fudan University Shanghai Cancer Center) between January 1, 2008 and December 31, 2010. Among them, 169 patients had the tumors located in the cervical or upper thoracic esophagus. Patients were staged according to the American Joint Commission (AJCC) 6th edition criteria. Initial staging consisted of a history and medical examination, routine blood tests, endoscopy, esophageal barium radiography and a chest computed tomography (CT). Sixty- two patients had PET-CT before treatments. However, 98 patients can’t afford it, and the other 9 patients declined PET-CT for no reason. Informed consent was obtained from each patient. This study was conducted in line with the guidelines of the World Medical Association and Declaration of Helsinki. The ethics committee of the Institutional Review Board of Shanghai Cancer Center approved the study. Details of failure patterns were documented from pre- and post-treatment CT, PET/CT scans, or endoscopy.

### Patient characteristic

From January 1, 2008 to December 31, 2010, a total of 169 patients with cervical and upper esophageal SCC were selected from 350 consecutive patients. The baseline characteristics of the 169 patients were shown in Table 1. One-hundred and twenty-eight patients were male and 41were female. The median age was 60 years old (range: 37–90 years). Eighteen patients had cervical esophageal SCC, and 151 had upper thoracic esophageal SCC. Eighty-two patients presented with Stage I-II disease, and 87 were Stage III-IV. The tumor lengths in long axis measured in barium esophagogram were <5 cm in 70 cases, 5–10 cm in 83 and >10 cm in 16. Seventy patients (41%) received ENI, and 99 patients (59%) did not. The baseline patient characteristics were balanced between the ENI group and the IFI group (Table 1).

**Table 1:** **Patients’ characteristics**

**Characteristics**	**IFI (%)**	**ENI (%)**	**P value**
**Sex**			
**Male**	74 (75%)	54 (77%)	0.679
**Female**	25 (25%)	16 (23%)	
**TNM stage**			
**I-II**	49 (49%)	33 (47%)	0.509
**III-IV**	50 (51%)	37 (53)	
**Median age (year)**	62	58	0.574
**Tumor length**			
**<5 cm**	43 (43%)	27 (39%)	0.416
**5-10 cm**	47 (47%)	36 (51%)	
**>10 cm**	9 (10%)	7 (10%)	
**Tumor location**			
**cervical esophagus**	9 (10%)	9 (13%)	0.480
**upper thoracic**	90 (90%)	61 (87%)	
**Nodal status**			
**N0**	42 (42%)	22 (32%)	
**Local nodes**	40 (40%)	31 (44%)	
**Distal nodes**	17 (18%)	17 (24%)	0.288

### Radiotherapy

All patients underwent CT simulation in the supine position, with CT images obtained at a 5-mm or 8-mm thickness throughout the entire neck and thorax. The GTV and the PTV were defined as followed. According to different PTV delineations, all the patients were divided into two groups: the ENI group and the IFI group.

Irradiation plan of IFI: The gross tumor volume (GTV) was defined as any primary tumors shown on the CT, or barium esophagogram, and mediastinal lymph nodes with the short axis of >10 mm and the cervical lymph nodes with the short axis of > =10 mm. For the planning target volume (PTV), a 1 cm margin was added around GTV, but 3 cm margins in the esophageal long axis superiorly and inferiorly to encompass potential submucosal invasions.

Irradiation plan of ENI: The definition for GTV was the same as that of IFI. The planning target volume (PTV) enclosed GTV and clinical target volume (CTV) which covered supraclavicular area with the upper margin at the caudal edge of cricoid cartilage, inferior margin at the sternal notch.

The CT data were registered in the treatment planning system (Pinnacle). Radiotherapy was delivered using 6 MV photons with multi-leaf collimator (MLC). To verify the field coverage, before treatment, the field size and position were confirmed by re-simulation. Portal images were done by an electron portal image device (EPID) to ensure the correctness of field and patient position.

Plan optimization was based on the dose-volume histogram (DVH), as follows: (1) the prescribed isodose curve covered 95% of the PTV; (2)the 95% isodose curve covered 99% of the PTV.(3) the maximum dose within the PTV was not allowed to exceed 110% of the prescribed dose. The dose constraints to the organs at risk were as follows: (1) the mean lung dose (MLD) was ≤15 Gy, and the V20 was ≤30%; (2) the mean heart dose (MHD) was ≤30 Gy; and (3) the maximum spinal cord dose was ≤45 Gy.

Doses to primary lesion and metastatic nodes were 60–68.4Gy, and 50.4-54Gy for elective node irradiation. The choice of ENI or IFI was physician dependent.

### Chemotherapy regimen

One hundred and forty-nine patients had PF regimen (cisplatin and 5-fluorouracil), 17 TP (taxane and cisplatin), and 3 TPF (taxane, cisplatin and 5-fluorouracil).

### Definition of failure patterns

**Figure 1:**
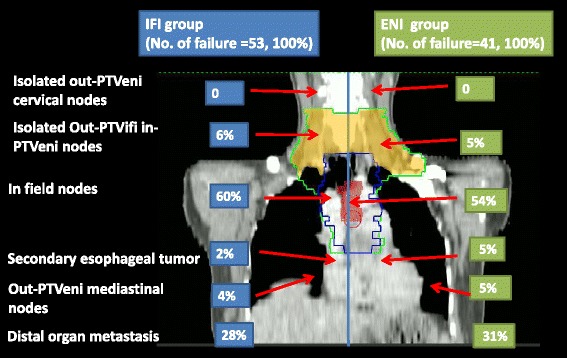
**Failure patterns of the IFI group and the ENI group.** ENI = elective nodal irradiation. Red: GTV; Blue: IFI field in the coronal direction; Green: ENI field in the coronal direction; Orange: the Out-PTVifi in-PTVeni field.

“PTVifi” means the PTV in IFI mode (Blue line in Figure 1).

“PTVeni” means the PTV in ENI mode (Green line in Figure 1).

“In-PTVifi recurrence” was referred to esophageal and/or regional lymph node failures within PTVifi.

“Out-PTVifi in-PTVeni metastasis” was referred to as lymph nodes metastasis occurred in the cervical prophylactic field of PTVeni thus out of PTVifi (Orange area in Figure 1).

“Out-PTVeni metastasis” was referred to lymph nodes metastasis occurred out of PTVeni.

The failures were confirmed by histology or cytology, or evidences of radiology including PET/CT. The confirmation of cervical nodes metastasis during the follow-up time relied on the biopsy and histology. We also considered different failures within 6 months as simultaneous failures.

### Toxicity

Treatment-related acute toxicity was scored by the RTOG criteria, including both acute and late injuries in either esophagus or lung. The observation started from the first day of treatment until death or the last follow-up visit.

### Statistics

Overall survival rate (OS), locoregional recurrence rate, distant metastasis rate and the incidence of lymph nodes metastasis were the primary endpoints. The Chi-square tests were used to compare the baseline characteristics between the two groups. The Kaplan-Meier methods were used to estimate these endpoints. Log-rank tests were used to compare the overall survival curves. The parameters were analyzed with respect to OS using the univariate and the multivariate analysis of Cox regression, including age, gender, TNM stage, nodal status, tumor length, ENI or IFI. All analysis was performed using SPSS 16.0 (SPSS Inc, Chicago, IL). The level of significance was set as *p* <0.05.

## Results

### Survival

Patients were followed until death or from 14 months to 59 months (median, 30 months) in those alive at last evaluation on December 31th, 2012. At the last follow-up visit, 76 patients (45%) were still alive. The 3-year OS rate for the entire group was 48%, and the median OS time was 27 months (95% Confidence Interval: 21–33 months). Three-year OS for the IFI group and the ENI group were 49% and 47%, respectively (*p =* 0.741). In multivariate analysis, TNM stage (*p* < 0.001), nodal status (*p* = 0.020) and tumor length (*p* = 0.036) independently predicted OS.

### Failure patterns

Failure patterns for the entire group were demonstrated in Table 2 and Figure 1. No significant difference was found between the IFI group and the ENI group regarding in field recurrences (*p* = 0.866) and distal organ metastasis (*p* = 0.728).

**Table 2:** **Failure patterns of IFI group and ENI group**

	**IFI (n = 99, 100%)**	**ENI (n = 70, 100%)**
Failure	53 (53%)	41 (59%)
In field recurrence	32 (32%)	21 (30%)
Alone	27 (27%)	17 (24%)
+ out-PTVeni cervical nodes	1 (1%)	1 (1%)
+ out-PTVifi in-PTVeni nodes	4 (4%)	3 (4%)
Isolated out-PTVifi in-PTVeni nodes	3 (3%)	2 (3%)
Isolated out-PTVeni cervical nodes	0	0
Out-PTVeni mediastinal nodes	2 (2%)	2 (3%)
Alone	0	0
+ out-PTVifi in PTVeni nodes	0	1 (1%)
+ with other distal nodes	2 (2%)	1 (1%)
Distal organ metastasis	15 (15%)	14 (20%)
Alone	8 (8%)	7 (10%)
+ in field recurrence	6 (6%)	5 (7%)
+ out-PTVifi in-PTVeni nodes	1 (1%)	2 (3%)
Secondary esophageal tumor	1 (1%)	2 (3%)
Alone	1 (1%)	1 (1%)
+ out-PTVifi in-PTVeni nodes	0	1 (1%)

Isolated out-PTVifi in-PTVeni cervical nodes metastasis occurred in 3 patients (3%) in the IFI group and 2 patients (3%) in the ENI group. Out-PTVifi in-PTVeni cervical nodes metastasis simultaneously with other failures happened in 5 patients (5%) in the IFI group and also 5 patients (7%) in the ENI group, respectively. Collectively, there were a total of 8 patients (8%) in the IFI group with out-PTVifi in-PTVeni cervical nodes progress, and 7 patients (10%) in the ENI group with these failures.

**Figure 2:**
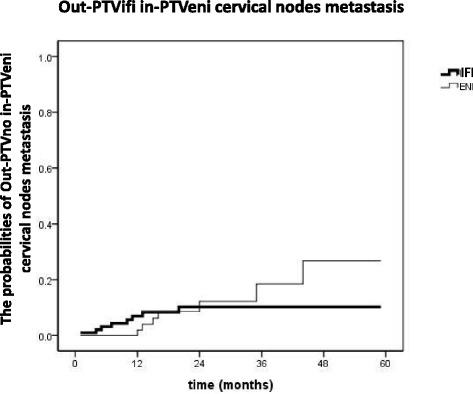
**Probability of Out-PTVifi in-PTVeni cervical nodes metastasis in the ENI group and the IFI group.**

The detailed conditions, further progressions and salvage treatments for the 15 patients were demonstrated in Table 3, and the probabilities of out-PTVifi in-PTVeni cervical nodes metastasis were showed in Figure 2. No significant difference was found between the IFI group and the ENI group in the rate of out-PTVifi in-PTVeni cervical nodes metastasis (*p =* 0.741). However, we found that for the patients in the IFI group, all the out-PTVifi in-PTVeni metastatic cervical nodes occurred within 2 years after treatments. However, for the patients in the ENI group, out-PTVifi in-PTVeni cervical nodes metastasis happened gradually since one year after treatment.

**Table 3:** **Out-PTVifi in-PTVeni cervical nodes metastasis**

**Patients no.**	**Radiation**	**Disease progress**	**Time to progress (months)**	**Next site**	**Salvage treatment**
**1**	ENI	Cervical nodes	13	Brain metastasis	BSC
**2**	ENI	Cervical nodes and lung metastasis	15	Esophageal progress	BSC
**3**	ENI	Cervical nodes and in-field recurrence	12	In-field nodes recurrence	BSC
**4**	ENI	Cervical nodes	35	Multiple metastasis	Chemotherapy
**5**	ENI	Cervical nodes and esophagus	16	Esophageal progress and other nodes metastasis	Target therapy and palliative radiotherapy
**6**	ENI	Cervical nodes and other nodes metastasis	44	Multiple metastasis	Chemotherapy
**7**	ENI	Cervical nodes and esophagus	24	Esophageal progress	BSC
**8**	IFI	Cervical nodes and esophagus	4	Mediastinal nodes	BSC
**9**	IFI	Cervical nodes	5	Pharynx cancer	Radiotherapy and chemotherapy
**10**	IFI	Cervical and other nodes	20	Multiple nodes metastasis	Radiotherapy
**11**	IFI	Cervical nodes and esophagus	10	Mediastinal nodes	BSC
**12**	IFI	Cervical nodes and esophagus	1	Mediastinal and celiac nodes and bone metastasis	BSC
**13**	IFI	Cervical nodes	11	Esophageal tumor progress	Radiotherapy
**14**	IFI	Cervical nodes	13	Multiple nodes metastasis	Chemoradiotherapy
**15**	IFI	Cervical nodes and esophagus	7	Esophageal progress	Surgery and radiotherapy

In addition, among those patients who had out-PTVifi in-PTVeni cervical nodes metastasis, only 1 patient (14%) in the ENI group received salvage radiotherapy after the failure. However, 5 patients (63%) in the IFI group had salvage radiotherapy.

### Toxicity

There were 6 patients (6%) in the IFI group and 4 patients (6%) in the ENI group experiencing grade ≥ 3 treatment-related esophageal injuries (p > 0.05). Grade 3 acute esophagitis were observed in 8 patients, 5 in the IFI group and 3 in the ENI group. Grade 4 acute esophagitis were observed in 2 patients, one in the IFI group and the other in the ENI group. Grade 3 late esophageal injuries were exhibited in 3 patients, 1 in the IFI group and 2 in the ENI group. Grade 4 late esophageal injuries were exhibited in one patient in the IFI group.

Grade ≥ 3 treatment-related lung injury occurred in 2 patients (2%) in the IFI group and 3 patients (4%) in the ENI group, respectively (p > 0.05). Grade 3 acute radiation induced pneumonitis was observed in 4 patients, 2 in the IFI group and 2 in the ENI group. Grade 3 radiation induced lung fibrosis was exhibited in one patient in the ENI group. Grade 4 radiation induced lung injury was not found.

## Discussion

The necessity of ENI for esophageal cancer has always been controversial [5-10], especially for ENI of the low neck. In the 1980s, regardless of tumor locations, the radiation field covered from supraclavicular region down to the joint region between esophagus and stomach [2]. However, involved field irradiation (IFI) has been used more frequently in the past decade [11]. We reported the outcome of 53 esophageal SCC who underwent definitive concurrent chemoradiation with IFI. We found that the median OS time was 30 months and 85% of patients failed locoregionally inside the irradiation fields, suggesting the un-necessity of ENI [5]. Yamashita also found that ENI was not helpful in the improvement of OS, and it brought higher rate of treatment-related deaths [6]. Zhang XL et al. performed a retrospective study on the failure patterns of 80 patients who underwent IFI, and found that the solitary regional nodal failure of out-of-field was acceptable for them [9]. However, ENI reduced the rates of distant lymph nodes metastasis in Hsu FM et al.’s study [10]. Onozawa M. et al. also showed ENI was effective for preventing regional nodal failure [12]. In Amini’s study, the celiac axis acts was considered as a “gateway” to the abdomen, and therefore, the coverage of celiac axis nodes was recommended, particularly for distal esophageal tumors [13]. For the cervical and upper thoracic esophageal cancers, it was speculated logically that ENI of low neck would be worthwhile due to the network of lymphatic communication. Chen JQ et al. reported that of the 1850 patients with thoracic esophageal SCC who underwent surgery, 1081 (58.4%) developed lymph nodes metastasis. The lymphatic metastatic rates were 35.6% for the cervical nodes. For the upper esophageal SCC, the most common node metastasis was in the cervical (49.5%) [14]. However,few reports have been published in the literature with regards to the comparison of detailed failure patterns between ENI and IFI.

In our retrospective study, 169 cervical and upper thoracic esophageal SCC were irradiated with or without ENI. OS, in-field recurrence incidence and distal organ metastasis rate were quite similar between the two groups. ENI did not reduce the cervical node progression rates. Ten percent of the patients in the ENI group had out-PTVifi in-PTVeni nodes metastasis, and 8% patients in the IFI group had such failures.

Moreover, we also found that for the patients in the IFI group, all the out-PTVifi in-PTVeni occurred within 2 years after treatment.In contrast, for patients in the ENI group, these failures happened gradually since one year after treatment. Therefore, our results supported that elective irradiation of low neck might not be able to decrease the rate of cervical nodes progression in the elective field. It could only delay these failures. In addition to that, earlier onset of cervical nodes progression in the elective field had no influence to both further failures (Table 3) and OS. On the contrary, it might bring higher rates of treatment related esophageal toxicity. Though no significant increase of esophageal toxicity was found from our data, Yamashita’s [6] and Ma’s studies [15] both found a statistically significant increase of radiation-induced side-effects and radiation-related death in esophageal SCC patients who underwent ENI.

From another perspective, due to previous cervical prophylactic radiation, only one patient in the ENI group underwent palliative radiotherapy after nodes metastasis in the out-PTVifi in-PTVeni field. However, in the IFI group, 63% of patients received salvage cervical radiation after these failures. More treatment opportunities after disease progression may contribute to the better prognostic result of patients in the IFI group.

In conclusion, ENI did not significantly improve OS and long-term control of cervical nodes in cervical and upper thoracic esophageal SCC patients. However, it is a retrospective study and included a small number of patients. Also it is a limitation that not all the patients had PET/CT, for PET/CT is not covered by insurance in China and many patients could not afford the expense. Therefore, this finding should be validated in further prospective studies.

## Abbreviations

AJCC: American Joint Commission
CTV: Clinical target volume
ENI: Elective nodes irradiation
GTV: Gross tumor volume
IFI: Involved field irradiation
OS: Overall survival
PTV: Planning target volume
SCC: Squamous cell carcinoma
